# Clinical Applications of Esophageal Impedance Monitoring and High-Resolution Manometry

**DOI:** 10.1007/s11894-012-0253-9

**Published:** 2012-02-21

**Authors:** Boudewijn F. Kessing, André J. P. M. Smout, Albert J. Bredenoord

**Affiliations:** Department of Gastroenterology and Hepatology, Academic Medical Center Amsterdam, Meibergdreef 9, 1105 AZ Amsterdam, The Netherlands

**Keywords:** Esophagus, Motility, Impedance, IIM, High-resolution manometry, Manometry, HRM, HREPT, Gastroesophageal reflux disease, Eosinophilic esophagitis, Fundoplication, Achalasia, Functional gastrointestinal disorder, Rumination syndrome, Adjustable gastric band, LABG, Belching, Aerophagia, Non-obstructive dysphagia, Children

## Abstract

Esophageal impedance monitoring and high-resolution manometry (HRM) are useful tools in the diagnostic work-up of patients with upper gastrointestinal complaints. Impedance monitoring increases the diagnostic yield for gastroesophageal reflux disease in adults and children and has become the gold standard in the diagnostic work-up of reflux symptoms. Its role in the work-up for belching disorders and rumination seems promising. HRM is superior to other diagnostic tools for the evaluation of achalasia and contributes to a more specific classification of esophageal disorders in patients with non-obstructive dysphagia. The role of HRM in patients with dysphagia after laparoscopic placement of an adjustable gastric band seems promising. Future studies will further determine the clinical implications of the new insights which have been acquired with these techniques. This review aims to describe the clinical applications of impedance monitoring and HRM.

## Introduction

**Fig. 1:**
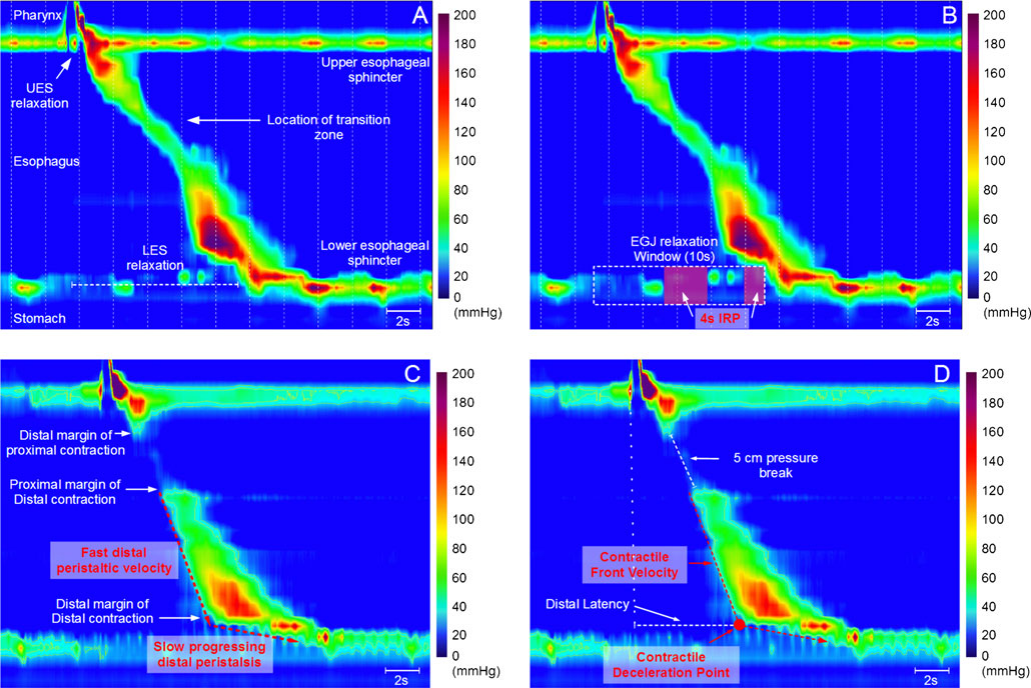
HRM plots of the esophagus. **a** Anatomical landmarks which can be identified with the use of HRM. **b** Assessment of LES relaxation using the integrated relaxation pressure (IRP). **c** Identification of peristaltic landmarks using the 20-mmHg and 30-mmHg isobaric contour lines. **d** Assessment of peristaltic function

Esophageal manometry is being performed in humans since the early 1950s [1]. Since then, esophageal manometry has greatly increased our understanding of esophageal function and is currently a widely performed technique to assess esophageal function. Conventional manometry assemblies detect pressure using a catheter with several water-perfused sideholes and with or without the addition of a, so called, sleeve sensor or solid state pressure transducers [2]. However, conventional manometry catheters are limited by gaps between the pressure sensors which are several centimeters long. To overcome this limitation, manometry catheters with smaller spacings between sideholes were developed, the so called high-resolution manometry (HRM) catheters. The current HRM catheters are no longer water-perfused but are equipped with intraluminal pressure transducers. With HRM, the clinician can simultaneously measure from hypopharynx to stomach which renders time-consuming pull-through techniques obsolete. Although measuring pressure at more levels provides more information, interpreting this many signals can be challenging. Therefore, esophageal pressure topography was adopted for the presentation of HRM data [3] (Fig. 1a). This technique assigns color to specific pressure levels which are than presented in a spatiotemporal plot. These pressure topography plots are more intuitive and easier learned by clinicians [4].

Johnson and deMeester introduced ambulatory pH-monitoring for the detection of reflux episodes in 1975 [5]. Since its introduction, pH-metry has become a commonly used technique for the evaluation of patients with symptoms suggestive of GERD. In 1991, impedance monitoring was introduced as a new technique to detect flow of fluids and gas through hollow viscera [6]. Esophageal impedance monitoring is based on the concept of measuring the resistance/impedance of an alternating electric current which is generated between pairs of electrodes mounted on a non-conductive catheter. When the esophagus is empty the catheter is in contact with the collapsed walls thus forming the medium between the sensors, this level is referred to as the baseline impedance level. The conductivity of fluids such as saline or gastric juice is high and the impedance level decreases if these substances form the medium between the electrodes. The conductivity of air is almost infinitely low which results in a high impedance if the medium between the electrodes consists of air. Placement of a series of electrodes along the catheter also enables one to evaluate the direction and velocity in which the gaseous or liquid medium is transported through the esophagus. Therefore, with esophageal impedance monitoring, the nature and movement of a substance in the esophagus can be detected.

The concept of a simultaneous measurement of reflux episodes and pressure dates back to the late 1950s when it was described by Tuttle and Grossman [7]. More recently, the technique of HRM and impedance monitoring have also been combined. Although assemblies consisting of a separate HRM catheter and an impedance catheter can be used, more recently, catheters in which these techniques are combined into a single catheter have become commercially available. Furthermore, the acquired impedance data can be visualized as color plots which are projected over the pressure topography plots acquired by HRM.

This review aims to describe the clinical applications of esophageal impedance monitoring and HRM. Furthermore, we aim to stipulate novel insights which have emerged from using the application of techniques.

## Clinical Applications of High-Resolution Manometry

### Interpretation of Data

When HRM was introduced in clinical practice, the Spechler and Castell classification of esophageal disorders was the gold standard for assessing manometric data [8]. However, this classification is based on conventional manometric data and it soon became clear that it fell short in the analysis of the complex data-set which is acquired by HRM. Therefore, specific criteria for the interpretation of HRM were developed [9]. This resulted in the so-called “Chicago classification,” named after the city in which these criteria were developed.

With HRM several new features were added to the analysis of esophageal pressure data. The integrated relaxation pressure (IRP) is a more complex measurement of deglutitive esophagogastric junction (EGJ) relaxation than the end-expiratory nadir pressure [10] (Fig. 1b). Conceptually, the IRP is the lowest average pressure for 4 contiguous or non-contiguous seconds during deglutitive EGJ relaxation [11]. This measure of deglutitive EGJ relaxation exhibited 98% sensitivity and 96% specificity for distinguishing well defined achalasia patients from control subjects and patients with other diagnoses [10].

Abnormalities of the force of the distal esophageal contraction can be classified as hypotensive, absent or hypertensive peristalsis. Hypotensive or absent peristalsis can be easily recognized using the isobaric contour lines (Fig. 1d). The threshold for hypotensive sections, also known as peristaltic pressure breaks, was recently classified as breaks >2 cm (20-mmHg isobaric contour) or 3 cm in length (30-mmHg isobaric contour) [18•]. The distal esophageal contraction is further characterized for the vigor of contraction using a newly developed measure, the distal contractile integral (DCI). The DCI integrates the length, contractile vigor, and duration of contraction of the distal esophageal segment contraction [11].

The first feature which is used to determine the transmission of peristalsis is the contractile front velocity (CFV) (Fig. 1d). Conceptually, the CFV is calculated from the slope of the line connecting the proximal margin of distal contraction and the distal margin of the distal contraction (Fig. 1c). A second feature of peristaltic transmission is the so-called contractile deceleration point which was recently proposed by Pandolfino et al. [13] (Fig. 1d). Conceptually, the contractile deceleration point demarcates the point at which the initial fast distal peristaltic velocity ends and the subsequent slow progressing distal peristalsis commences. A third feature of peristaltic transmission is the distal latency which determines the timing of the contractile deceleration point relative to the swallow [12] (Fig. 1d).

A recent study by Pandolfino et al. aimed to apply the CFV and distal latency to refine the diagnosis of distal esophageal spasm [14]. These authors observed that a large heterogeneous group of patients can be identified as having distal esophageal spasm based on the criterion of simultaneous contraction. However, most of the patients with simultaneous contractions do not have a clinical presentation suggestive of esophageal spasm [14]. Therefore, the authors proposed that only esophageal spasms characterized by a short distal latency and spastic achalasia are considered as pathological [14]. Future studies will determine whether this new classification will result in different clinical outcomes.

The novel manometric criteria defined by the Chicago Classification could potentially result in a decreased reproducibility. However, Bogte et al. found that these novel HRM criteria yield reproducible results [15]. Furthermore, the reproducibility of LES resting and relaxation pressure assessed with HRM is better than with conventional manometry.

Below, we will discuss new insights gained with high-resolution manometry.

### Non-obstructive Dysphagia

Fox et al. described that the close spacing of pressure sensors at the HRM catheter allows the clinician to identify isolated hypotensive segments in the peristaltic contraction [16]. These isolated hypotensive segments are caused by a delay and/or spatial gap between the contraction of the striated muscle in the proximal esophagus and the initiation of the smooth muscle in the distal esophagus. This region in the esophagus is commonly referred to as the transition zone [17]. With the use of concurrent video-fluoroscopy it was demonstrated that the presence of a hypotensive segment can predict the success of bolus transport [16]. Moreover, a hypotensive segment >5 cm in length is uniformly associated with incomplete bolus clearance [18•]. Whereas transition zone defects are far less common than distal peristaltic abnormalities or abnormalities at the EGJ they may be related to dysphagia in a minority of patients [17].

The close spacing of pressure sensors at the HRM catheter also allows one to differentiate between the pressure generated by the lower esophageal sphincter (LES) and the crural diaphragm if a hiatal hernia is present. This spatial separation of the LES and the crural diaphragm in patients with a hiatal hernia is observed as a double high-pressure zone compared to a single high-pressure zone in subjects without a hiatal hernia [19]. Scherer et al. demonstrated that, in a hiatus hernia patient, EGJ obstruction is not always caused by the LES but can also be caused by dysrelaxation of the crural diaphragm [20]. However, whether this information changes therapeutic outcome needs to be determined by future studies.

The diagnostic work-up for non-obstructive dysphagia can be troublesome due to the lack of agreement between objective measurements of esophageal function and the lack of bolus transit assessment as measured by conventional manometry and subjective perception of bolus passage [21•]. This suggests that increased bolus passage perception in patients without mechanical obstruction might be due to esophageal hypersensitivity thereby limiting the use of esophageal function tests using conventional manometry in patients with non-obstructive dysphagia [21•]. However, the clinical application of HRM has resulted in a more specific classification of esophageal disorders and future research will determine whether this has clinical implications.

### Achalasia

Ghosh et al. described that with the use of HRM substantial shortening of the esophagus can be observed in achalasia patients [10]. If conventional manometry is used in these patients the movement of the EGJ between pressure sensors can result in a pseudorelaxation. This suggests that the use of HRM may result in an improved sensitivity for the diagnosis of achalasia when compared to conventional manometry [10].

Furthermore, HRM has prompted the recognition of three different subtypes of achalasia which are achalasia with minimal esophageal pressurization (type I, classic), achalasia with esophageal compression (type II) and achalasia with spasm (type III) [22]. However, it can be argued that these subtypes can also be recognized with conventional manometry.

With the use of intraluminal ultrasonography a longitudinal muscle contraction of the distal esophagus was recently identified as the cause of pan-esophageal pressurization in type II achalasia [23]. Furthermore, esophageal emptying occurs intermittently during periods of pan-esophageal pressurization [23]. Patients with achalasia of types I and III have no emptying or relatively normal emptying during most swallows, respectively [23]. This suggests that, in achalasia patients, esophageal emptying results from swallow-induced longitudinal muscle contraction of the distal esophagus, which increases esophageal pressure and allows flow across the non-relaxed EGJ [23].

The clinical relevance of identifying the achalasia subtype was demonstrated by a recent study by Pratap et al. who demonstrated that patients with type II achalasia respond best to treatment followed by type I and type III [24].

### Bariatric Surgery

Laparoscopic placement of an adjustable gastric band (LAGB) is a widely performed treatment for obesity. However, dysphagia, vomiting, and regurgitation are common side effects of gastric banding. Several studies have aimed to assess esophageal motility and clearance in symptomatic LAGB patients using high resolution manometry [25, 26]. By comparing symptomatic patients with successfully treated patients, relevant parameters such as intra-bolus pressure and lower esophageal contractile segment have been identified [25, 26]. It has been suggested that these parameters can be used to determine whether the LAGB should be removed [26]. However, whether HRM can replace the barium swallow, which is the current gold standard to determine flow across the gastric band, cannot be concluded on the basis of the available literature. Furthermore, it is also not known whether a pre-surgical HRM can predict treatment outcome. Therefore, although the use of HRM looks promising, future studies are warranted to determine its clinical application in patients undergoing gastric banding or in patients with side effects of LAGB placement.

### Eosinophilic Esophagitis

A recent study by Roman et al. evaluated the use of HRM in 48 patients with eosinophilic esophagitis [27]. Using high-resolution manometry, 37% of patients with eosinophilic esophagitis were classified as having abnormal esophageal motility. Pan-esophageal pressurization was present in 17% of patients with eosinophilic esophagitis and 2% of GERD patients while compartmentalized pressurization was present in 19% of patients with eosinophilic esophagitis. In theory, pan-esophageal pressurization in patients with eosinophilic esophagitis could be the result of decreased compliance of the esophagus. These patterns were not seen in control subjects. Although motility disorders were more frequent in patients with eosinophilic esophagitis than in controls, the prevalence and type were similar to those observed in GERD patients [27]. These results suggest that there is no clinical indication for HRM in patients with eosinophilic esophagitis.

## Clinical Applications of pH-Impedance Monitoring

### Reflux Disease

**Fig. 2:**
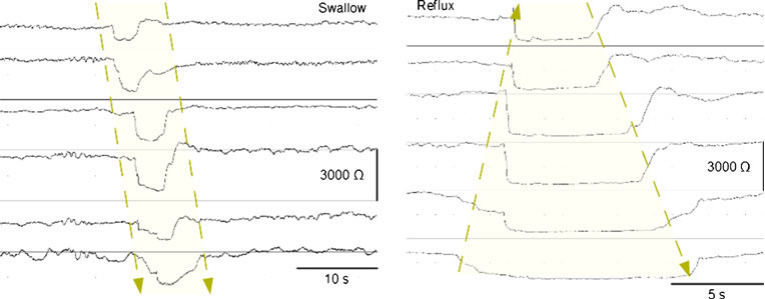
Impedance tracings of a wet swallow and a liquid reflux episode. Wet swallows are characterized by an antegrade drop in impedance channels whereas a reflux episode is characterized by a retrograde drop in impedance channels after which a swallows clears the refluxate and the impedance levels return to their respective baseline level

By far the largest application of esophageal impedance monitoring in clinical practice is in the diagnostic work-up of patients with GERD symptoms. Impedance monitoring detects retrograde flow in the esophagus whereas pH monitoring detects reflux events as a drop in the pH-level (Fig. 2). Compared to impedance monitoring, detection of reflux with pH monitoring is clearly inferior [28]. This is mainly attributable to the detection of weakly acidic reflux by impedance monitoring since impedance measures fluid flow instead of change in pH. Therefore, with the use of impedance monitoring, the clinician can correlate an increased number of reflux symptoms with a reflux episode [29, 30]. The latter increases the diagnostic yield in patients with GERD and shows that the use of pH-metry alone will result in an underestimation of GERD and an overestimation of functional dyspepsia and functional heartburn [29, 30]. Therefore, the use of combined pH-impedance monitoring is currently considered as the gold standard for the detection of reflux episodes and is becoming available in an increasing number of centers.

Hemmink et al. assessed whether cessation of PPI could further increase the diagnostic yield in patients with GERD [31]. These authors found that the best chance to assess a relationship between symptoms and reflux episodes is after cessation of PPI. Therefore, these authors concluded that ambulatory pH-impedance monitoring should preferably be performed after cessation of PPI therapy [31]. However, a different study by Pritchett and colleagues suggested that impedance-pH on PPI may be best to study refractory [57]. Recent guidelines therefore suggest that the choice of on-PPI or off-PPI should rely heavily on the pretest probability of GERD and the question that needs to be answered [58]. Obviously, more research is warranted in this area to provide a definitive algorithm for reflux monitoring in GERD.

Anti-reflux surgery, also known as surgical fundoplication, is a widely performed intervention for GERD. Besides decreasing the number of acid reflux episodes, anti-reflux surgery also reduces the number of weakly acidic reflux episodes [32]. Therefore, one could suggest that GERD patients with symptoms due to weakly acidic reflux episodes are also suitable candidates for anti-reflux surgery. This would favor pH-impedance monitoring over pH-metry in the pre-operative work up of GERD patients.

Despite the fact that reflux monitoring is a valuable diagnostic tool, evidence for the use of pH-metry as a preoperative predictor of surgical outcome has shown conflicting evidence [33]. Furthermore, the quality and consistency of the data on the use of pre-operative reflux monitoring are mixed and the strength of the associations remains unclear [33]. Moreover, the Symptom Association Probability (SAP), which is a measure for the correlation between symptoms and reflux episodes has not been shown to be a preoperative predictor of surgical outcomes if measured by pH-metry [34]. However, studies which specifically assessed the role of pH-impedance monitoring as a preoperative predictor of surgical outcome have not yet been performed. Therefore, although pH-impedance monitoring is the gold standard for the detection of reflux episodes, it remains unclear whether pre-operative pH-impedance monitoring is better than pH-metry alone.

Between reflux episodes and swallows, the esophageal lumen is collapsed and the resulting baseline impedance level is determined by the esophageal wall. Several studies hypothesized that impedance baseline measurements could also be used to evaluate changes in esophageal mucosa integrity [35, 36, 37•]. Farré et al. demonstrated that baseline impedance values remains lower after perfusion with acid in rabbits [37•]. Furthermore, a positive correlation was found between the transepithelial resistance of esophageal mucosa and baseline impedance levels [37•]. A study from our center showed a negative correlation between the acid exposure time in the esophagus and the baseline impedance levels [35]. Furthermore, even when the acid exposure time was in the physiological range, GERD patients were characterized by lower baseline impedance levels than controls [35]. Moreover, PPI can increase low baseline impedance levels in adults and children [35, 36]. These findings suggest that baseline impedance is related to esophageal acid exposure and could be a marker of reflux-induced changes to the esophageal mucosa. Future studies are warranted to determine the clinical relevance of the baseline impedance level.

Whereas the detection of reflux episodes with pH-metry can be easily detected as a pH-drop <4, the detection of reflux episodes with impedance monitoring can be somewhat more troublesome. Several studies have assessed inter-observer variability for the detection of reflux episodes with impedance monitoring in the same center, however, a multi-center study has not yet been performed in adults. A recent multi-center study by Loots et al. assessed inter observer variability in impedance tracings of children and found only moderate agreement between the observers [38]. A different approach which could potentially decrease inter-observer variability is the use of an automatic analysis. The results from studies which assessed the clinical use of the automatic analysis show that this is a helpful tool which shows a high correlation with a manual analysis [38, 39].

### Reflux in Pediatric Patients

During the work-up of reflux symptoms in children pH-impedance monitoring is increasingly being performed. Studies revealed that a significant proportion of all reflux episodes is weakly acidic and weakly acidic reflux episodes are more prevalent than acid reflux episodes in infants with symptoms [46, 47•]. Therefore, pH-impedance monitoring increases the diagnostic yield for GERD in children and is superior to pH-metry [48].

A recent study by Rosen et al. aimed to determine predictors of fundoplication outcome in children using pH-impedance monitoring [49]. These authors found that no single reflux marker predicted fundoplication outcome. Furthermore, neither a positive symptom index nor a positive symptom sensitivity index predicted postoperative improvement. These results suggest that pH-impedance monitoring in children may not be a useful tool in predicting fundoplication outcome [49].

### Belching Disorders and Aerophagia

Gastric belching is a physiological mechanism which enables venting of gas from the stomach to the esophagus in order to prevent accumulation of excess gas in the stomach or duodenum [40, 41]. With the use of esophageal impedance monitoring a second mechanism of belching was identified in 2004, the so-called supragastric belch [42]. During a supragastric belch, air is rapidly sucked into the esophagus and is immediately followed by a rapid expulsion of air without ever reaching the stomach.

The clinical relevance of this differentiation between supragastric belches and gastric belches was demonstrated in patients with excessive belching as their main symptom [42]. Bredenoord et al. demonstrated that these patients are characterized by an increased frequency of supragastric belches but not of gastric belches [42]. Moreover, a pilot study in patients with excessive supragastric belching revealed that speech therapy could decrease the severity of belching symptoms [43].

Belching is a common symptom in GERD patients with an incidence of 40% to 49%. Hemmink et al. showed that supragastric belching can also occur in patients with GERD [44]. Moreover, supragastric belching is associated with troublesome belching symptoms in GERD patients. In theory, supragastric belches could offer more specific treatment options for GERD patients with troublesome belching symptoms. Hemmink et al. also observed that supragastric belches can precede reflux episodes and suggested that supragastric belches could induce reflux episodes [44]. Studies which assess the role of speech therapy in this specific group of GERD patients are currently being performed in our center.

Aerophagia is a disorder which is characterized by abdominal bloating and abdominal distension due to an excessive volume of intestinal gas [45]. With the use of impedance monitoring an increased amount of air swallows was identified as a possible cause of the excessive intestinal gas. This finding could, in theory, offer more specific treatment targets and future studies will determine the clinical implications of this finding.

With the use of impedance monitoring the clinician can differentiate between supragastric belching, aerophagia and gastric belching in patients with gas related symptoms such as belching and abdominal bloating. This results in a more accurate diagnosis and could possibly result in a more specific treatment for selected patients.

## Clinical Applications of Combined High-Resolution Manometry and pH-Impedance Monitoring

### Rumination Syndrome

The rumination syndrome is a functional gastroduodenal disorder of unknown etiology characterized by persistent or recurrent regurgitation of recently ingested food into the mouth. Diagnosis of rumination is currently based on clinical features as defined by the Rome III criteria [50]. The pathophysiology is incompletely understood, but involves a rise in intra-gastric pressure, generated by a voluntary, but often unintentional, contraction of the abdominal wall musculature causing retrograde movement of gastric contents into the esophagus [51]. A large proportion of regurgitation episodes in patients with the rumination syndrome are weakly acidic, therefore, pH-impedance monitoring is superior to pH-metry for the detection of regurgitation episodes in rumination patients [52]. Furthermore, with the use of combined manometry-impedance monitoring Rommel et al. could differentiate between belching and rumination [53]. Although the number of patients with the rumination syndrome who are studied by HRM is still relatively small, the first results suggest that HRM results in a more accurate detection of rumination episodes [52]. Therefore, in case of diagnostic uncertainty, manometric evaluation combined with pH-impedance monitoring may confirm the diagnosis. Whether HRM is superior to conventional manometry in the diagnostic work-up of the rumination syndrome needs to be determined by future studies.

### Non-obstructive Dysphagia

Combined manometry-impedance can be used to identify esophageal function abnormalities in patients with non-obstructive dysphagia [54]. The advantage of combined HRM-impedance over conventional manometry is the ability to detect incomplete bolus clearance and combine this information with a detailed assessment of peristalsis [12]. With the use of combined HRM-impedance it was shown that incomplete bolus clearance occurs with failed peristalsis or with pressure breaks in the peristaltic contraction [12] (Fig. 1d). Moreover, pressure breaks in the peristaltic contraction are more frequent in patients with dysphagia compared to control subjects [12].

A recent study by Burgess et al. applied this technique of combined HRM-impedance monitoring and described that little further information was gained compared to HRM alone [55]. However, studies that assess the clinical relevance of combined HRM and impedance monitoring with a focus on bolus escape at the transition zone have not yet been performed. Therefore, the currently available literature does not support a clear clinical application of combined HRM-impedance monitoring in patients with non-obstructive dysphagia at this moment.

## Discussion

The application of HRM has improved our understanding of the esophagus and increases the diagnostic yield of manometry. Furthermore, the design of the manometry assembly and the presentation of the acquired data are easier to perform in clinical practice and easier learned by the clinician.

The technique of HRM, the interpretation of the acquired data and the classification of disease is constantly being improved. Recently, 3D-HRM was developed which measures pressure with several radial pressure sensors at the location of the EGJ [56]. 3D-HRM is currently only being used in research and their clinical applications need to be further explored.

pH-impedance monitoring has proven to be a diagnostic tool which is superior to pH-metry in the diagnostic work-up of adult and pediatric patients with GERD symptoms. Furthermore, the application of pH-impedance monitoring with regard to belching complaints has already lead to more specific treatment targets. Currently, evolving concepts regarding the use of baseline impedance levels and the role of supragastric belching in patients with GERD are being assessed. The outcomes of these studies will determine the clinical relevance of these novel concepts and could expand the clinical applications of impedance monitoring.

## Conclusions

HRM and pH-impedance monitoring have lead to an improvement in diagnostics and classification of esophageal disorders. Future studies will further determine the clinical implications of the new insights which have been acquired with these techniques.
